# Maintaining and Improving Psychological Well-Being

**DOI:** 10.1097/jnr.0000000000000333

**Published:** 2019-05-20

**Authors:** Yea-Ing Lotus SHYU

Psychological well-being is very important for individuals whether they are living in the community, at work, or dealing with a crisis in life. In working to improve psychological well-being, in addition to preventing and treating mental and behavioral disorders, efforts are needed to reduce common environmental or mental stresses. While nurses are in a unique position to monitor and prevent psychological problems such as depression and anxiety in vulnerable populations, their own psychological well-being and the stressful situations that they regularly face impact greatly on their work performance.

Many of the nine articles in this issue focus on identifying predictors of psychological problems in high risk and vulnerable populations. Two articles explore depression in older persons, one describes the experiences of mothers living through prenatal loss, one explores the mediating role of alcohol in the relationship between anxiety and poor sleep, and one explores the predictors of caregiver burden for caregivers who care for bedridden patients. An additional two articles focus on the effects of psychological well-being on nursing performance, including one that examines how work-related quality of life influences organizational commitment in prison nurses and one that explores nurse-related stress, work violence, and burnout. The studies in this issue were conducted in Turkey, Korea, Taiwan and Saudi Arabia, highlighting the similarity in concerns across different countries and the importance of the issue of psychological well-being globally.

Findings of these studies may have valuable clinical and practice implications as well as increase the knowledge base of nursing. The contributions of these researchers are greatly appreciated.

